# Brains Vs Bowels

**Published:** 1875-04

**Authors:** Ben. H. Pratt


### THE BISTOURY.

Vol. XI. ELMIRA, N. Y., APRIL., 1875. No. 1.

### Contributions.

### BRAINS VERSUS BOWELS.

BEN. H. PRATT.

It is natural to be proud of those whom we love —natural to wish that they for whom we entertain great admiration should make a good impression upon those whose good opinion we desire to cultivate. Hence it is that parents, whose love for their own children is so unexceptional that they who do not exhibit it are rated as monsters, are anxious that their offspring should appear to others as pretty and bright and cunning and smart and cute as they do to themselves. This sentiment is well nigh universal, and although it is prompted by one of the most beautiful emotions exhibited by the race, parental love, still, in its ulterior workings it has a pernicious effect upon the future welfare of the child.

It is a pleasing fancy, possessed by every parent of an ordinarily endowed child, that it is somewhat of a prodigy—that it is a little more "knowing" than other people's pets—that it is showing unmistakable evidences of being talented in one way or another—that germs of genius are now and then peeping out of its daily life—that it certainly promises something wonderful in the future if these germs are only coaxed, cared for, nourished, encouraged, developed. Thus, oftentimes, the parent, in the great anxiety to find some such evidence of genius, snaps up a very little circumstance and builds thereupon a wondrous airy castle. The mother is particularily prone to see manifestations of this sort, and in her neighborly chats will very solemnly reveal her fondly cherished beliefs to her coterie of intimate friends. Then follow the usual expressions of astonishment at the revelation; the eyes open wider—the brow lifts—the lips move in smothered you-don't-say-sos—curiosity is at once agog to see the child, referred to in such mysterious whisperings. Then, may be, the prodigy is introduced, and a number of catechetical questions propounded to it; when, more than likely, the obstinate little thing declines to "show off," and makes of itself a very disagreeable, pouty, cross, ill-mannered little imp, and is hustled off to the nursery, bawling and kicking its objections in a most ungenius-like manner. But possibly the child may have been so well drilled in previous displays of its remarkable attainment, that it may go through the whole manual without a miss, and gallantly sustain its reputation. In this case there follow the usual pettings and huggings and kissings and adulatory praises, until the child begins to believe itself something more than ordinary, and exhibits toward those of its age an air of immense superiority. It vaunts itself a genius, and the neighborhood is full of astonishing stories about the wonderful sayings and doings of "that boy of Smith's," or of "that girl of Brown's,"—in fact, the child is pampered into the impression that all it has to do is to grow up and become great.

This is most unfortunate. It effectually kills effort. It don't do to have children too smart. Had we a choice, we'd much prefer being the bawling, kicking, wiggling, tow-headed brat that is carried forth in disgrace from the "showing off," than be the pert, self-satisfied "mother's pride" that owns the reputation of being a prodigy. The former invaribly turns out better—makes a better man or woman—becomes more self-reliant—don't disgust people with its exhibitions of conceit—is more tractable and approachable—more willing to be taught—isn't dictatorial—don't have to be taken down in its efforts to overbear and brow-beat—is beloved, while the other is often most cordially hated.

Just here, then, it seems to us that parents would show more good sense if, in the rearing of their children, they would not make so much ado over any little outcropping of what they may suppose to be the wrestling of genius within the juvenile mind. If it's there, let it be—it will keep. If it isn't there, but only imagined, you won't spoil the child by making it believe it is there. We wouldn't advise a parent to discourage the natural bent of a child's genius, if he really exhibits such, nor even to make light of it; but the hot-house system of developing one talent at the expense of every other cannot be too earnestly discouraged.

Again, the daily drill which some children are obliged to go through, is absolutely stultifying in its effects. It is the custom, with many parents, to impress upon their little ones the necessity of learning their letters and becoming proficients in spelling and reading at an age when the tax upon the brain required for the mastery of these accomplishments is greater than it can bear. Daily lessons, occupying a specified time, and insisted upon against the inclinations of the very young child, are harmful. The parent will admit, upon reflection, that it is not necessary for the child's education that it be thus early taught, but the same pride that suggests development in other respects, goads on to this. To have people know how smart your child is—to tell how it knew all its letters when two years old—how it could read very well at four—how it had read such and such books through at six—this is mere brag, and that is all there is of it. It seems cruel, when we are reminded of what tears have been shed—what slappings have been administered—what punishments have been resorted to to accomplish all this, to say nothing of the permanent injury done to the mental faculties of the child. Perhaps many little ones may be so strong brained, and so precociously apt, as not to be injuriously affected by such treatment; but if this be so the evidence of it will be plain. If Johnny begs "to say his letters," hear him say them until he's tired of it, and not a moment longer. Don't set him brain tasks during the time which nature is plainly reserving for physical development. If even up to the age of six years he evinces no taste for study, what matters it?—he will make it up in the end. Brain power, between six and eight years old, is sufficiently ample, if it has not been tampered with, to accomplish in six months all that the forced brain has achieved in the preceding four years. Recall your school days. Don't you remember how some big, ignorant lout from the country came to the Academy, and how you smart town chaps snickered at the ridiculous blunders he made at first? Don't you remember, too, how that by and by he caught up to you, and pretty soon, when you had a hard sum to do, the teacher sent you to him to have it solved? Didn't you wonder then how he got to know so much in so short a time? These humiliations are still fresh in our memory, and are instances in proof of our theory that the physical should be first well developed, regardless of the intellectual, and then the mental power acquired will master learning with a rapidity, and an ease, and an understanding, that will surprise those who have been stuffing and cramming their weak heads from infancy up, and have, after all, only a superficial knowledege of their acquirements.

Drilling children for the purpose of "showing them off" is, we claim, doing them a foolish, permanent and criminal injury, and we are prepared, upon the authority of those who have made insanity and mental debility, together with the causes leading thereto, a study, to say that nearly thirty-three per cent. of such cases have been traced back to two causes, viz: the forcing of the brain in very early youth beyond its capacity to sustain such pressure, and the necessity of using the brain when the physical health was such as to prove of no assistance in recuperating that organ after its overstrained efforts.

The tendency of the age is to develop one faculty at the expense of all others. New methods of teaching—ingeniously designed traps to catch the ambition of the child, and tempt him to essay those acquirements which are beyond his years—the eternal ding dong of parental reproach at all times sounded into the ears of our little ones, because, forsooth, they are not quite so far advanced in their accomplishments as neighbor Soandso's boy or girl—humiliating such by invidious comparisons that discourage the child—these are excessively unwise and reveal the very sad truth that parents are really more solicitous for the present ability of their children to make a good "show-off" than they are to have them grow up mentally and physically well balanced. The strong bodied idiot is not more to be pitied than the puny prodigy of learning. The fear of being a dunce has ruined many a strong constitution, and we always feel rejoiced, in these days of hot-house education, when we find a boy that has so little sensitiveness as to blurt right out his indifference as to whether he be at the head or foot of his class so long as he can whollop his chum; or a girl that has independence enough to disregard the sneers of those who can say their tables through without a mistake, when she knows that she can beat the biggest girl in school at jumping rope, and can take her own part in a snow-balling bout with any boy of her age.

Let us give our children good lungs, good strong arms, good legs, good red blood—let them acquire endurance, wind, muscle, red cheeks and clear complexions—in a word, give them a good physical start; then will they acquire such ambitions and such power of applying their mental strength, that they'll overtake all their companions in the long run, and come to the goal of manhood and womanhood, and enter upon its duties and struggles and trials, fresh and strong, while their feebler brethren and sisters are dosing for debilities, and wrestling in the agonies of dyspepsia and bad livers.

